# Antibody-Mediated Oligodendrocyte Remyelination Promotes Axon Health in Progressive Demyelinating Disease

**DOI:** 10.1007/s12035-015-9436-3

**Published:** 2015-09-26

**Authors:** Bharath Wootla, Aleksandar Denic, Jens O. Watzlawik, Arthur E. Warrington, Moses Rodriguez

**Affiliations:** 1 Departments of Neurology, Mayo Clinic, 200 1st Street SW, Rochester, MN 55905 USA; 2 Departments of Immunology, Mayo Clinic, 200 1st Street SW, Rochester, MN 55905 USA; 3 Mayo Clinic Center for Multiple Sclerosis and Autoimmune Neurology, Mayo Clinic, 200 First Street SW, Rochester, MN 55905 USA; 4 Center for Regenerative Medicine, Neuroregeneration, Mayo Clinic, 200 First Street SW, Rochester, MN 55905 USA

**Keywords:** Antibody, Remyelination, Multiple sclerosis, Theiler’s murine encephalomyelitis virus, Magnetic resonance spectroscopy, *N*-acetyl-aspartate, Brainstem, Axons, Protection

## Abstract

Demyelination underlies early neurological symptoms in multiple sclerosis (MS); however, axonal damage is considered critical for permanent chronic deficits. The precise mechanisms by which axonal injury occurs in MS are unclear; one hypothesis is the absence or failure of remyelination, suggesting that promoting remyelination may protect axons from death. This report provides direct evidence that promoting oligodendrocyte remyelination protects axons and maintains transport function. Persistent Theiler’s virus infection of Swiss Jim Lambert (SJL)/J mice was used as a model of MS to assess the effects of remyelination on axonal injury following demyelination in the spinal cord. Remyelination was induced using an oligodendrocyte/myelin-specific recombinant human monoclonal IgM, rHIgM22. The antibody is endowed with strong anti-apoptotic and pro-proliferative effects on oligodendrocyte progenitor cells. We used ^1^H-magnetic resonance spectroscopy (MRS) at the brainstem to measure *N*-acetyl-aspartate (NAA) as a surrogate of neuronal health and spinal cord integrity. We found increased brainstem NAA concentrations at 5 weeks post-treatment with rHIgM22, which remained stable out to 10 weeks. Detailed spinal cord morphology studies revealed enhanced remyelination in the rHIgM22-treated group but not in the isotype control antibody- or saline-treated groups. Importantly, we found rHIgM22-mediated remyelination protected small- and medium-caliber mid-thoracic spinal cord axons from damage despite similar demyelination and inflammation across all experimental groups. The most direct confirmation of remyelination-mediated protection of descending neurons was an improvement in retrograde transport. Treatment with rHIgM22 significantly increased the number of retrograde-labeled neurons in the brainstem, indicating that preserved axons are functionally competent. This is direct validation that remyelination preserves spinal cord axons and protects functional axon integrity.

## Introduction

The conduction of electrical impulses through long axonal segments of the neuron is essential for proper functioning of the central nervous system (CNS). Oligodendrocytes produce myelin [1], which ensheath axons and integrally support in fast axonal conduction. In multiple sclerosis (MS), a chronic inflammatory demyelination of the CNS, conduction block in demyelinated axons is responsible for early neurological deficits, while axonal transection and accumulating neuronal loss underlie permanent disability [2, 3]. Endogenous myelin repair (remyelination) occurs spontaneously in early lesions and restores secure conduction [3]; however, this process is hampered as disease advances [4]. In experimental models, induction of oligodendrocyte apoptosis causes demyelination, myelin splitting, and/or vacuolization resulting in motor pathologies of variable severity [5–7]. In chronic rat experimental autoimmune encephalomyelitis (EAE), axon loss in the spinal cord determined permanent neurological disability [8]. Demyelinated axons are vulnerable to further degeneration/transection [9] as the mutual supply of trophic and metabolic support between oligodendrocytes and the underlying axons is lost. This suggests that repairing persistent demyelination may ameliorate clinical deterioration and may decrease axonal degeneration. In support of this concept, Kornek et al. showed that remyelinated MS shadow plaques had significantly less evidence of axonal injury compared to inactive demyelinated lesions [10]. Likewise, Totoiu et al. showed an association of axon sparing and a recovery of function after transplant-mediated remyelination in the mouse hepatitis virus-induced model of demyelination [11]. Therefore, strategies to promote remyelination either by transplantation of exogenous progenitors or targeting endogenous ones to enhance remyelination may protect axons. Our laboratory developed a recombinant human monoclonal IgM antibody, rHIgM22, which binds specifically to live oligodendrocytes and myelin and promotes remyelination in mouse models of MS [12–14]. This IgM is a true human natural antibody and belongs to the normal human germline immunoglobulin repertoire [15, 16]. rHIgM22 is self-reactive, but is non-toxic even at 4000 times the therapeutic dose [14]. rHIgM22 recently completed a 16-site phase I clinical trial in patients with MS with complete safety [17].

Based on epidemiological evidence, viral infections are thought to play a role in the initiation and/or exacerbation of MS [18, 19]. Theiler’s murine encephalomyelitis virus (TMEV), a single-stranded RNA virus belonging to the *Picornaviridae* family, induces inflammatory demyelinating disease in the spinal cord of susceptible strains of mice following infection [20]. TMEV-induced demyelinating disease (TMEV-IDD) is a natural chronic-progressive CNS demyelinating disease of susceptible strains of mice, with similarities to primary progressive MS (reviewed in [21]). Demyelination in association with an intense inflammatory response begins in the spinal cord around day 21 following infection and is well established by day 45. Demyelination in infected mice continues to worsen until approximately 90–100 days post-infection (dpi), then reaches a plateau [22]. Retrograde labeling studies of demyelinated spinal cord axons performed in our laboratory [23] revealed a reduction in labeled neuron cell bodies in the brainstem that indicated death or dysfunction. Magnetic resonance spectroscopy (MRS) is a non-invasive tool to measure and quantify tissue metabolites. *N*-Acetyl-aspartate (NAA), a derivative of aspartic acid, is found in high concentrations in all areas of the brain, but is undetectable in non-neuronal tissue. NAA is the second most abundant free amino acid metabolite in the nervous tissue after glutamate and provides a prominent peak in MRS readouts in vivo [24]. NAA levels are reduced under numerous neuropathological conditions such as brain injury, stroke, and Alzheimer’s disease. Reduced NAA concentrations also correlated with reduced axonal numbers in lesions of secondary progressive MS (SPMS) patients [25], primary progressive MS (PPMS), and relapsing remitting MS (RRMS) [26]. Therefore, NAA is a key metabolite marker for neuronal health, viability, and number [27]. Consequently, spontaneously remyelinating mouse strains recover brainstem NAA concentrations [28], whereas non-remyelinating mouse strains do not show recovery of NAA concentrations [29]. We demonstrated that MRS measurements of NAA at the brainstem reflects the integrity of many ascending and descending spinal cord pathways whose neuronal cell bodies lie in the brainstem, thus highlighting the use of brainstem NAA concentrations as a surrogate marker for spinal cord pathological changes, including demyelination and remyelination [29].

To date, there is no direct evidence that IgM-mediated remyelination protects demyelinated axons from degeneration in a progressive model of MS. To address this issue, we treated TMEV-infected Swiss Jim Lambert (SJL) mice with rHIgM22 or controls at 90 dpi to monitor axon loss. This time point was chosen because it is the beginning of axon loss, but without complete loss of axon function, such that recovery of axonal loss/function may occur. In addition, at 90 dpi, demyelination is evident and brainstem NAA concentrations are significantly lower compared to their baseline levels prior to infection [29]. We previously reported that the extent of remyelination induced by rHIgM22 reaches maximal levels by 5 weeks after treatment and remains out to 10 weeks [14]. By adopting this approach, we postulated that rHIgM22-mediated remyelination protects denuded axons from further destruction.

## Materials and Methods

### Ethics Statements

The Mayo Clinic Institutional Animal Care and Use Committee (IACUC) approved all animal protocols used in this study.

### Theiler’s Virus-Induced Demyelinating Disease Model (TMEV-IDD)

Six- to 8-week-old SJL/J (prototypic susceptible strain—H-2^s^) mice were purchased from the Jackson Laboratories (Bar Harbor, ME). Mice were anesthetized and intracerebrally injected at 8 weeks of age with 2 × 10^5^ plaque-forming units (p.f.u.) of the Daniel’s strain of TMEV in a 10-μl volume. This resulted in >98 % incidence of infection with rare fatalities. The clinical disease course of identically aged mice and treatment strategy are previously published [30, 31]. Mice in this study developed observable gait deficits by 90 days post-infection and were considered representative of the disease model.

### Antibody Treatment

SJL mice at 90 dpi were treated with a single 100-μg intraperitoneal dose of rHIgM22 (*N* = 11) or control IgM (*N* = 10) dissolved in phosphate buffered saline (PBS) or 500 μl of the vehicle, PBS (*N* = 11), alone. Uninfected mice (*N* = 3) were used as a reference for retrograde labeling studies (see below).

### Magnetic Resonance Spectroscopy

MRS was performed using a Bruker Avance 300 MHz (7T) vertical bore NMR spectrometer (Bruker Biospin, Billerica, MA). During data acquisition, animal core temperatures were maintained at 37 °C by a flow of warm air. Inhalational isoflurane anesthesia 1.5–2.5 % in oxygen was delivered via a nose cone. MRS data were obtained and quantified from a (2.5 × 2.5 × 2.5) mm^3^ voxel (15.625 μl), placed over the brainstem as reported previously [29]. The brainstem was chosen for study since many of the neuronal cell bodies of descending and ascending spinal cord axons are present at this site. MRS data was collected from each mouse before treatment and at 5 and 10 weeks later. The same investigator selected all voxels based on anatomical landmarks to maintain strict uniformity. Bruker’s VSEL sequence, an implementation of the standard PRESS sequence, was used for voxel-based spectroscopy, with built-in water suppression pulses.

### Spinal Cord Morphometry

We assessed spinal cord morphometry in all groups 10 weeks following treatment. Mice were anesthetized with sodium pentobarbital and perfused intracardially with Trump’s fixative (phosphate-buffered 4 % formaldehyde/1 % glutaraldehyde, pH 7.4). Spinal cords were removed and sectioned precisely into 1-mm blocks. In order to represent the samples along the length of the spinal cord, every third block was post-fixed, stained with osmium tetroxide, and embedded in araldite plastic (Polysciences, Warrington, PA). One-micron sections were cut and stained with 4 % *p*-phenylenediamine to visualize the myelin sheaths. We examined ten spinal cord cross sections, spanning the entire spinal cord from the cervical to the distal lumbar regions, from each mouse. Each spinal cord quadrant from every coronal section was graded for the presence of inflammation, demyelination, and remyelination. Areas of demyelination were characterized by naked axons, cellular infiltration, and macrophages with engulfed myelin debris. Abnormally thin myelin sheaths relative to axonal diameter and absence of Schwann cells are indicative of oligodendrocyte remyelination. Thick myelin sheaths and the one-to-one relationship between axons and Schwann cells identify Schwann cell remyelination. Areas of demyelination and remyelination are well demarcated and allow accurate quantitative assessment at ×10 and ×40 magnifications, respectively. Demyelination scores were expressed as the percentage of spinal cord quadrants examined with pathological abnormality. A maximum score of 100 indicated pathological abnormality in every quadrant of all spinal cord sections of a given mouse. A spinal cord quadrant was considered to be remyelinated if greater than 75 % of the area of the field contained remyelination. Remyelination scores were calculated as the ratio (%) of spinal cord quadrants with remyelination over the number of spinal cord quadrants with pathologic white matter abnormalities (demyelination), which had the potential to be remyelinated. All grading were performed on coded sections without knowledge of the experimental group.

To quantify myelinated axons, a mid-thoracic (T6) spinal cord section from each animal was examined. This level of the cord was chosen because it contains both ascending and descending axons with neuronal soma in the brainstem [32]. To ensure a uniform intensity of myelin labeling, all spinal cord T6 sections used in the study were stained with the same batch of 4 % para-phenylenediamine for exactly 20 min. An Olympus Provis AX70 microscope and a ×60 oil immersion objective were used to capture six sample areas of normal-appearing white matter without demyelination from each section. The six fields were collected in a clockwise manner around the section to obtain representative samplings of the posterior-lateral, antero-lateral, and anterior columns. Images were centered between the gray matter and meningeal surface. Approximately 400,000 μm^2^ of white matter was sampled from each mouse. Absolute myelinated axon numbers were measured using automated counting software that recognizes circular intact myelin sheathes and calculated as reported [29]. Data were represented as the absolute number of all axons sampled per mid-thoracic spinal cord section. All values were averaged per group.

### Brain Pathology

Brain pathology was assessed after the last MRS measurement, using our previously described technique [33]. Following perfusion with Trump’s fixative, we made two coronal cuts in the intact brain at the time of removal from the skull (one section through the optic chiasm and a second section through the infundibulum). We used the *Atlas of the Mouse Brain and Spinal Cord* corresponding to sections 220 and 350, page 6 [34], as a guide. This resulted in three blocks that were then embedded in paraffin. This allowed for systematic analysis of the pathology of the cortex, corpus callosum, hippocampus, brain stem, striatum, and cerebellum. Resulting sections were then stained with hematoxylin and eosin. Pathological scores were assigned without knowledge of experimental group to the different areas of the brain. Each area of the brain was graded on a five-point scale as follows: 0, no pathology; 1, no tissue destruction with only minimal inflammation; 2, early tissue destruction (loss of architecture) and moderate inflammation; 3, definite tissue destruction (demyelination, parenchymal damage, cell death, neurophagia, neuronal vacuolation); and 4, necrosis (complete loss of all tissue elements with associated cellular debris). Meningeal inflammation was assessed and graded as follows: 0, no inflammation; 1, one cell layer of inflammation; 2, two cell layers of inflammation; 3, three cell layers of inflammation; and 4, four or more cell layers of inflammation. The area with maximal tissue damage was used for assessment of each brain region.

### Retrograde Labeling

Retrograde labeling was performed on a separate cohort of mice (*N* = 23; uninfected = 3, rHIgM22 = 9, PBS = 11), 9 weeks after antibody treatment as described [23]. Seven days after surgery, mice were sacrificed, perfused with 4 % paraformaldehyde, and brains and spinal cords collected. Serial vibratome (Lancer Series 1200) sections (40 μm thick) of the brainstem were collected and mounted under Vectashield (Vector Laboratories Inc., Burlingame, CA). Cell bodies containing retrograde tracer (FluoroGold), visualized by UV illumination (360–370 nm excitation, 420–460 nm emission) were counted at ×200 from 16 brainstem slices that encompass the 2.5-mm brainstem voxel used to measure NAA concentrations. A cell was counted as positive if a large cross section of the cell body was labeled with FluoroGold. The number of labeled cell bodies in the brain stem nuclei reflected the number of healthy and functional axons in the spinal cord. All analyses were performed without knowledge of the experimental group.

### Graphs and Statistics

All graphs were plotted on GraphPad Prism v5.1 (GraphPad Software, Inc., La Jolla, CA, USA). Statistics were computed using SigmaPlot v11.0 (Systat Software, Inc., San Jose, CA, USA). Results were compared by two-tailed Student’s *t* test if normally distributed or by Mann–Whitney rank sum test if non-normally distributed. We used one-way ANOVA for comparing normally distributed data sets for more than two groups or Kruskal–Wallis ANOVA on Ranks if data was non-normally distributed. In all analyses, *p* < 0.05 was considered as statistically significant. Correlation coefficients between paired sets of data were determined using the Pearson product moment correlation. Fisher’s exact test was used to compare animals with improved NAA versus those with no change/decrease in NAA levels.

## Results

### A Single Peripheral Dose of rHIgM22 Administered at 90 Days Post-TMEV Infection Improves Brainstem NAA Concentrations at 5 and 10 Weeks Post-treatment

**Fig. 1:**
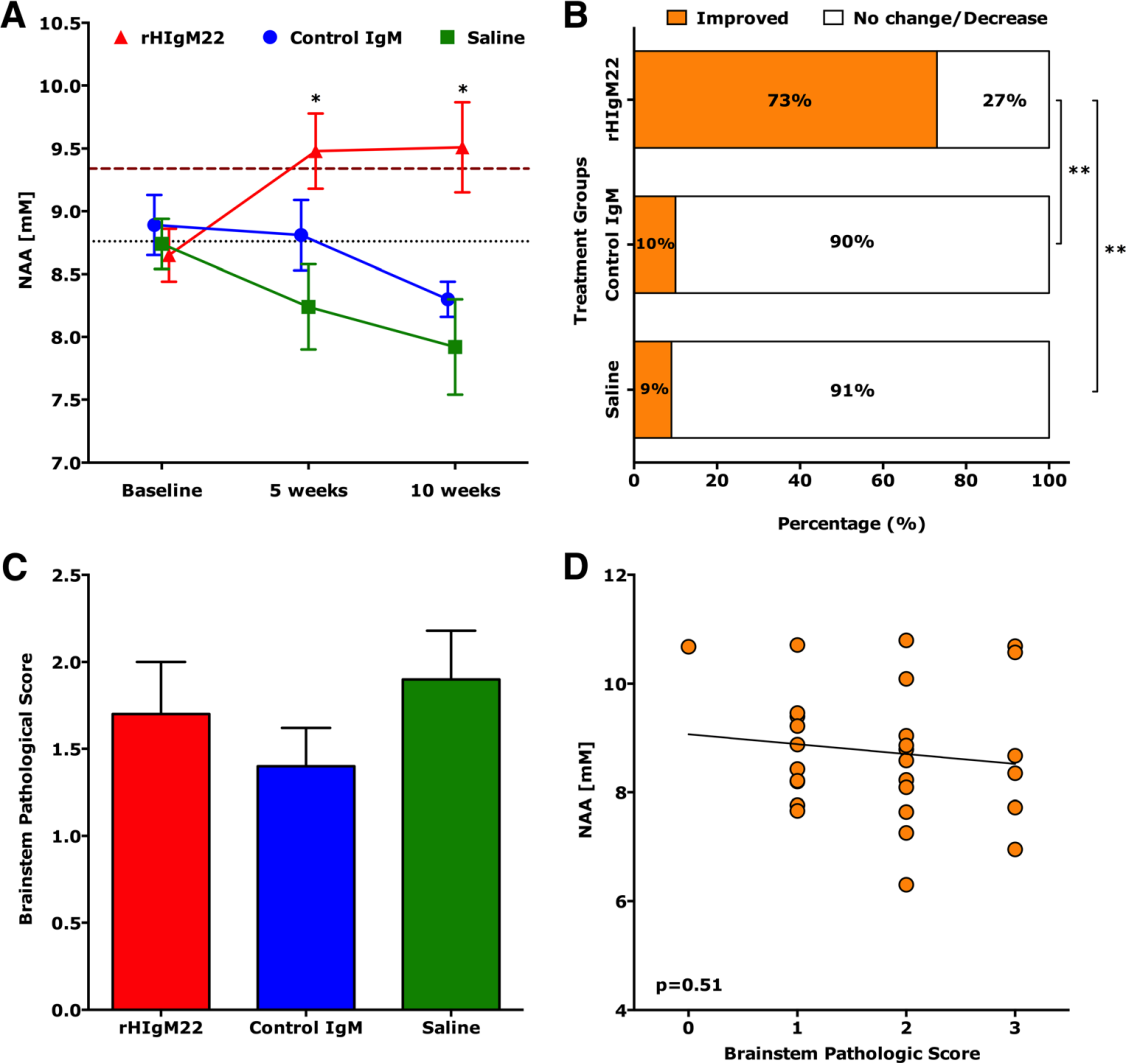
A single dose of rHIgM22 improves brainstem NAA concentrations. Brainstem MRS was collected from TMEV-infected SJL mice at 90 days post-infection prior to treatment, and at 5 and 10 weeks after treatment. **a** In the rHIgM22-treated group, NAA levels increased at 5 and 10 weeks to 9.51 mM (*p* = 0.025 and *p* = 0.04, respectively, one-way ANOVA repeated measures). In a saline-treated group, at 5 weeks, NAA concentrations dropped, and by 10 weeks were significantly lower (*p* = 0.077 and *p* = 0.012, respectively, one-way ANOVA repeated measures). In the control IgM-treated group, no significant difference in NAA concentrations (*p* = 0.17) was found. Initial NAA concentrations were not different across groups (*p* = 0.76, one-way ANOVA). The *thick-dashed cayenne colored line* represents the average baseline NAA concentrations from uninfected healthy mice (*N* = 6) and the *thin dotted black colored line* represents the average baseline NAA concentrations from all treatment groups. *Symbols* represent the treatment groups: rHIgM22 (*red triangles*), control IgM (*blue circles*), and saline (*green boxes*). **b** Changes in individual NAA concentrations were calculated at the 10-week point versus before treatment. NAA levels were considered improved if the difference (NAA_10 weeks_ − NAA_before_) was greater than or equal to two times the baseline SEM. NAA levels improved in 1 of 10 in the control IgM-treated group, 1 of 11 in the saline-treated group, and 8 of 11 in the rHIgM22-treated group. **c** Brainstem pathological scores (means ± SEM) were similar across treatment groups (*p* = 0.43). **d** The individual brainstem NAA concentration plotted against the brainstem pathological score showed no correlation (*p* = 0.51), *p < 0.005, one-way ANOVA

Brainstem NAA concentrations were evaluated as a measure of overall spinal cord neuronal health. Brainstem NAA concentrations in uninfected healthy mice were 9.34 ± 0.04 (mean ± SEM; *N* = 6). Following TMEV infection, mice were divided into three random groups and all have similar baseline NAA concentrations at 90 dpi. The natural human IgM, rHIgM22, enhances remyelination in TMEV-infected SJL mice [14]. To investigate whether antibody-mediated remyelination protects denuded axons from further destruction and improves brainstem NAA concentrations, we treated groups of TMEV-infected SJL mice at 90 dpi with a single 100-μg dose of rHIgM22 (*N* = 11), a non-CNS-reactive control IgM (*N* = 10) [35] or saline (*N* = 11). At 5 and 10 weeks post-treatment, NAA concentrations in the rHIgM22 were 9.48 ± 0.30 and 9.51 ± 0.36, respectively. Conversely, NAA concentrations in the control groups were 8.81 ± 0.28 (control IgM) and 8.24 ± 0.34 (saline) at 5 weeks post-treatment and 8.30 ± 0.14 (control IgM) and 7.92 ± 0.38 (saline) at 10 weeks post-treatment. Our results show that only the rHIgM22-treated mice exhibited a significant increase in NAA concentrations at later time points (*p* < 0.005, one-way ANOVA), whereas in saline and control IgM-treated mice, NAA concentrations remained unchanged at the 5-week point or decreased at the 10-week time point (Fig. 1a). Animals with improved NAA concentrations at 10 weeks after treatment were then compared to animals with no change/decrease in NAA (Fig. 1b). NAA concentrations were considered improved if the difference (NAA_10 weeks_ − NAA_baseline_) was greater than or equal to two times the baseline SEM. We found that 73 % (8 of 11 animals) of the rHIgM22-treated group showed improved concentrations of NAA compared to 10 % (1 of 10 animals) of the control IgM-treated group or 9 % (1 of 11 animals) of the saline-treated group. A Fisher’s exact test revealed the control IgM-treated group did not differ from the saline-treated group (*p* = 0.94), whereas the rHIgM22-treated group differed from both control groups (*p* = 0.008 and *p* = 0.008, respectively).

### Impact of Brain Pathology on rHIgM22-Mediated Improvement of Brainstem NAA Concentrations

To test the possibility that variability in brainstem pathology influenced NAA concentrations, we analyzed brainstem pathological scores across groups and found consistent minimal disease (Fig. 1c). In addition, individual NAA concentrations did not correlate with brainstem pathology (*p* = 0.51, Fig. 1d). Taken together, these results rule out the possibility that local brainstem pathology affects NAA concentration within the measured voxel.

### rHIgM22 Promotes CNS Remyelination in TMEV-IDD

**Fig. 2:**
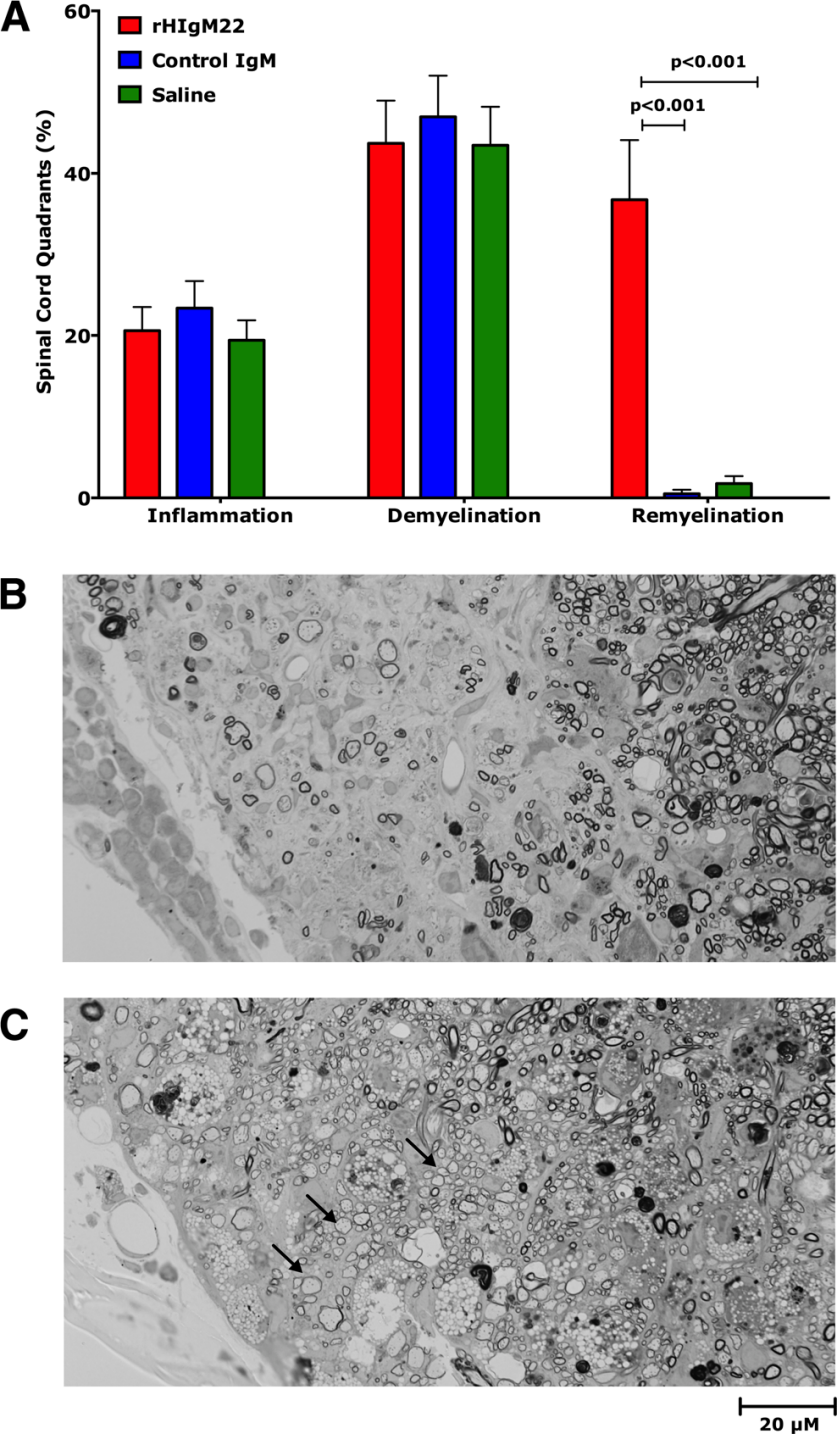
rHIgM22 promotes spinal cord remyelination. Data is expressed as a percentage of quadrants with the pathological abnormality as a function of all spinal cord quadrants examined. After the last MRS scan, 10 weeks post-treatment, mice were sacrificed, and spinal cords were removed and processed for morphologic analysis. **a** Mice from all three treatment groups showed similar levels of spinal cord inflammation and demyelination pathology. rHIgM22 treatment increased the extent of remyelination as compared to control groups (*p* < 0.001, one-way ANOVA). Typical images of demyelination (**b**) and remyelination (**c**) obtained from the antero-lateral spinal cord columns, areas with frequent disease, are shown. *Arrows* represent examples of remyelinated axons

Following the final MRS scan, spinal cords were removed, embedded in plastic, sectioned at 10 levels representing the entire cord, and stained with para-phenylenediamine to visualize myelin sheaths. Ten sections from each cord were scored for inflammation, demyelination, and remyelination. All treatment groups showed equivalent levels of inflammation and demyelination (Fig. 2a). However, only mice treated with rHIgM22 showed enhanced remyelination (Fig. 2a). Examples of typical demyelinated (Fig. 2b) and remyelinated (Fig. 2c) lesions are shown.

### rHIgM22 Treatment Preserves Spinal Cord Axons

**Fig. 3:**
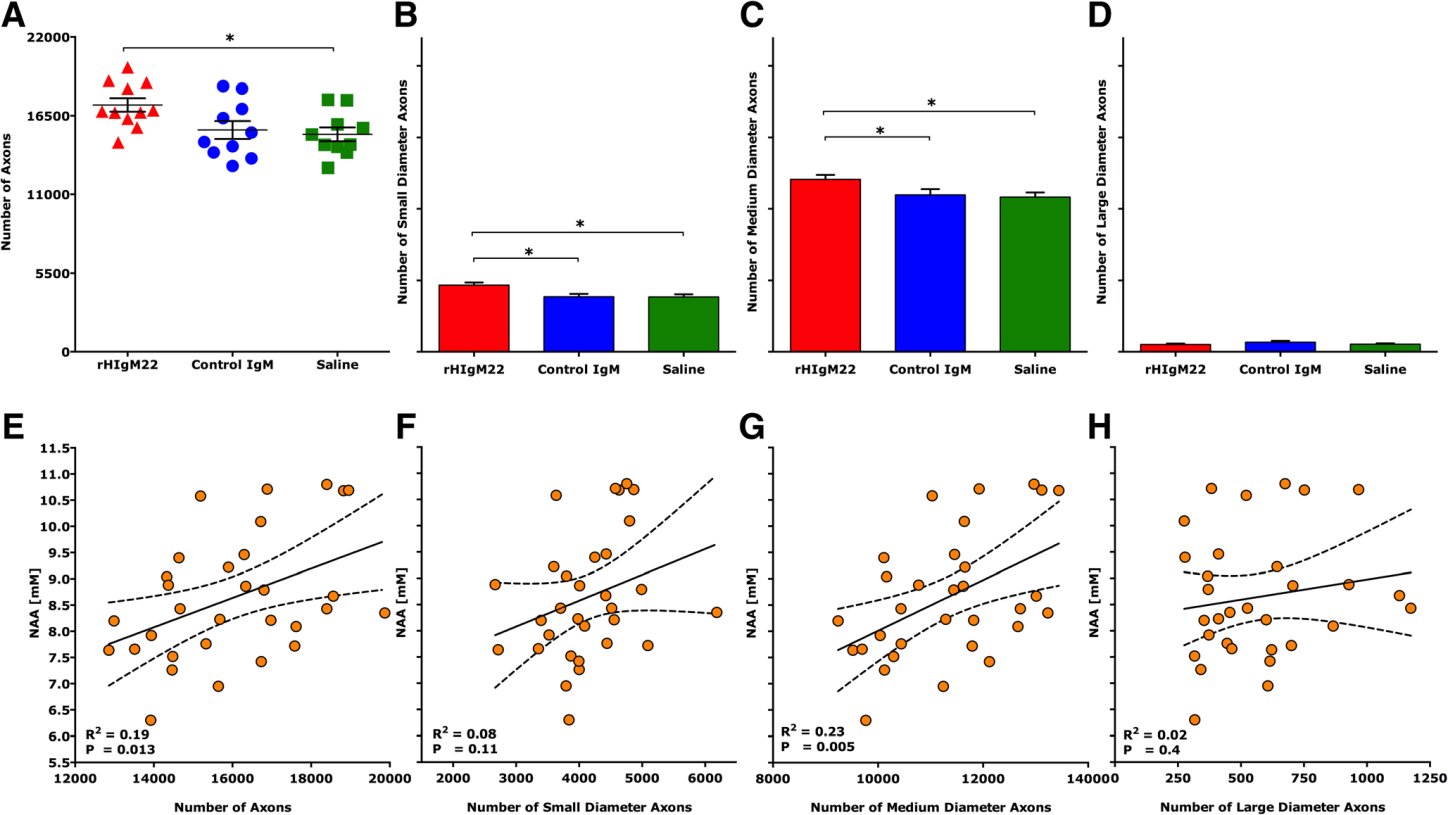
A single dose of rHIgM22 preserves axons in the spinal cord. When axons of all calibers were analyzed, a greater number were counted in the rHIgM22-treated group as compared to the saline-treated group (**a**). *Symbols* in **a** represent the treatment groups: rHIgM22 (*red triangles*), control IgM (*blue circles*), and saline (*green boxes*). Considering axons of different calibers, a greater number of **b** small-caliber (1–3.99 m^2^) and **c** medium-caliber (4–10 m^2^) axons were counted in the spinal cords of rHIgM22-treated mice. The number of **d** large-caliber axons (>10 m^2^) was not statistically different between groups of mice (*p* = 0.55). *Bars* represent the average of absolute number of myelinated axons ± SEM. **e** A positive correlation (*p* = 0.013, *R* ^2^ = 0.19) exists between brainstem NAA concentrations collected at the 10-week time point from all mice and the number of mid-thoracic level axons. Individual brainstem NAA concentrations are plotted against **f** small-caliber (*p* = 0.11, *R* ^2^ = 0.08), **g** medium-caliber (*p* = 0.005, *R* ^2^ = 0.23), and **h** large-caliber (*p* = 0.4, *R* ^2^ = 0.02) axons. *Dotted lines* represent 95 % confidence band of the best-fit linear regression line

We determined the number of myelinated axons from mid-thoracic (T6) spinal cord sections. Axon counts from uninfected mice averaged 21,285 ± 830 (mean ± SEM). As expected, TMEV-IDD resulted in axon loss at 10 weeks post-infection (Table 1). However, when the total number of mid-thoracic myelinated axons was compared across treatment groups, the rHIgM22-treated mice, with improved NAA concentrations, also contained more axons than control IgM- and saline-treated groups (Table 1) suggesting relative axon protection. We then performed a detailed analysis of axon distribution (Fig. 3a), which revealed a greater preservation of both small-caliber (1–3.99 m^2^, Fig. 3b) and medium-caliber (4–10 m^2^, Fig. 3c) axons in rHIgM22-treated mice, whereas large-caliber axons (>10 m^2^, Fig. 3d) were equivalent across all treatment groups (*p* = 0.55, one-way ANOVA). Furthermore, when we plotted individual brainstem NAA concentrations against the total number of mid-thoracic axons (Fig. 3e) and medium-caliber axons (Fig. 3g), we found a positive and statistically significant correlation (*R*^2^ = 0.19; *p* = 0.013 and *R*^2^ = 0.23; *p* = 0.005, respectively). We did not find significant correlations between NAA concentrations and small-caliber (Fig. 3f) or large-caliber (Fig. 3h) axons.

**Table 1:** A single dose of rHIgM22 protects axons in the spinal cord

Treatment	Average number of T6 axons^a^	Compared to saline	Compared to control IgM
rHIgM22 (*N* = 11)	17,254 ± 473	*p* = 0.007	*p* = 0.037
Control IgM (*N* = 10)	15,508 ± 627	*p* = 0.69	
Saline (*N* = 11)	15,198 ± 484		
Uninfected mice (*N* = 3)	21,285 ± 830		

*N* number of animals

^a^The average ± SEM of sampled myelinated axons per cross section

### rHIgM22 Preserves Axon Transport

**Fig. 4:**
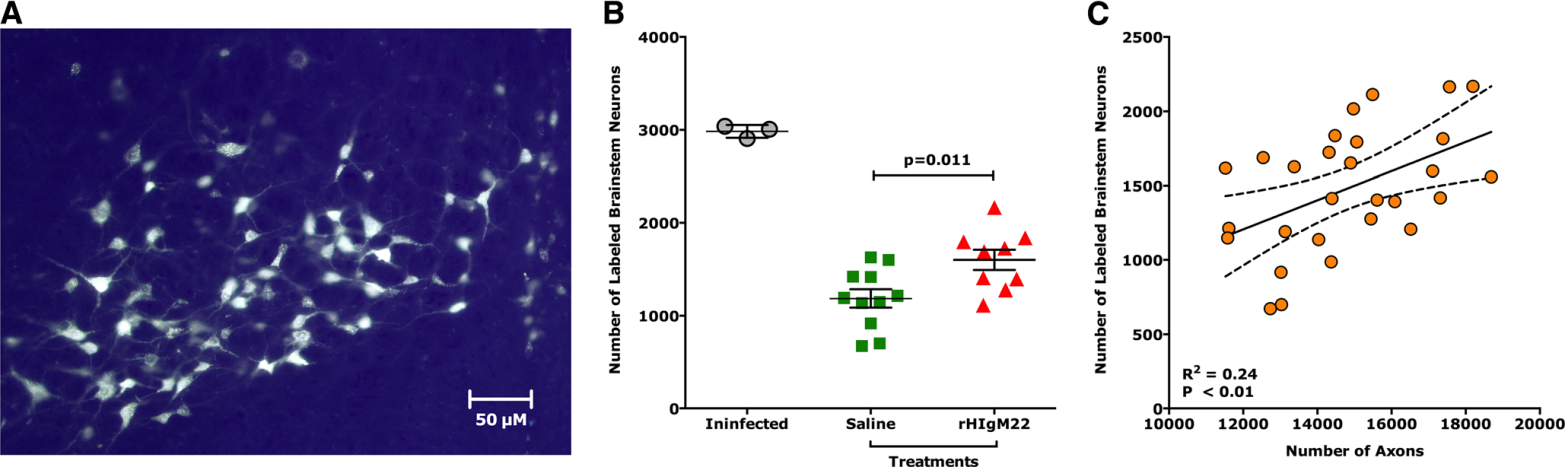
rHIgM22 improves number of retrograde-labeled brainstem neurons. FluoroGold-labeled neurons were counted in brainstem sections that correspond to the (2.5 × 2.5 × 2.5) mm^3^ voxel used for collecting MRS data. **a** Example of a cluster of fluorescently labeled neurons in the brainstem is shown. Extensive labeling of cell bodies as well as axons and dendrites can be easily appreciated. **b** The number of labeled neurons in the uninfected mice (*N* = 3) is shown as a reference. rHIgM22 treatment increased the number of retrograde-labeled brainstem neurons compared to the saline-treated group (*p* = 0.011, *t* test). The average number of retrograde-labeled brainstem neurons ± SEM per treatment group is shown. *Symbols* represent the treatment groups: rHIgM22 (*red triangles*) and saline (*green boxes*). **c** Number of fluorescently labeled neurons correlated positively and significantly to the number of axons (*p* = 0.008, *R* ^2^ = 0.24)

Retrograde labeling relies on both anatomically continuous axons and preserved retrograde transport mechanisms to provide an assessment of axonal integrity. Because MRS studies showed no differences between saline and control IgM-treated TMEV-infected mice, we studied only the saline treatment as a control group for these experiments. One week post-surgery (i.e., 10 weeks post-treatment), mice were sacrificed and brains and spinal cords were collected. As a reference, retrograde labeling was performed in age-matched uninfected mice. Figure 4a shows an example of a cluster of fluorescently labeled neurons in the brainstem, where cell bodies as well as dendrites and axons are clearly seen. For each descending neuron population, cell bodies containing retrogradely transported label were quantified. All uninfected mice (*N* = 3) had similar levels of labeling and presented with an average number of 2983 ± 39 (mean ± SEM) labeled brainstem neurons. We previously reported that retrograde labeling studies performed in demyelinated mice demonstrated a large reduction in fluorescently labeled neuron cell bodies in the brainstem [23]. Similarly, we found that the control-treated animals (*N* = 11, 1185 ± 98) and rHIgM22-treated animals (*N* = 9, 1599 ± 99) had fewer labeled neurons compared to uninfected mice. However, we found on an average more fluorescently labeled brainstem neurons in the rHIgM22 group compared to the saline group (*p* = 0.011, *t* test, two-tailed) (Fig. 4b). We again determined axons from the mid-thoracic (T6) spinal cord sections in this cohort. We found more axons in the rHIgM22-treated group that tended towards significance compared to the saline-treated group (15,137 ± 517 versus 13,758 ± 575, *p* = 0.07). When we plotted individual retrograde-labeled neurons as a function of quantified axons of all calibers from all animals, irrespective of the treatment groups (Fig. 4c), we found a positive and statistically significant correlation (*R*^2^ = 0.24; *p* = 0.008). Our results support the hypothesis that a single treatment with rHIgM22 promoted remyelination that directly protected spinal cord axons and indirectly neuronal cell bodies in the brainstem, which may underlie the increased brainstem NAA concentrations and enhanced retrograde labeling.

## Discussion

The aim of the current study was to investigate whether remyelination protects axons following myelin denudation. This was achieved by treating TMEV-infected mice demyelination with a remyelination-promoting human antibody, rHIgM22. Our results show that improvement of remyelination at 10 weeks post-treatment resulted in a significant increase of brainstem NAA concentration. This is consistent with our previous observations where higher brainstem NAA concentrations were measured in two different strains of mice that spontaneously remyelinate [28, 29]. In addition, we found that improved remyelination correlated with a greater preservation of spinal cord axons. This result strongly supports the concept that remyelination protects axons from death. The number of spinal cord axons correlated well with the corresponding increase in brainstem NAA concentrations. Our analysis of spinal cord axon distribution revealed that rHIgM22-mediated remyelination primarily protected medium-caliber axons, which correlated well with NAA concentrations in the brainstem. However, large-diameter axons were not protected. Possibly small- and medium-caliber axons were preferentially remyelinated in this model. Alternatively, large-diameter axons may shrink to become medium-diameter axons as a result of injury and protected from further injury by remyelination.

The most direct evaluation of descending axon integrity was retrograde labeling at the T6 level of the spinal cord with the fluorescent tracer FluoroGold. Our results show that demyelination in SJL mice is accompanied by a reduction of axonal transport as detected by a decrease in labeled brainstem neurons. The majority of demeylinated lesions in the TMEV-IDD occur at the cervical and thoracic levels. Therefore, a reduction in the number of labeled brainstem cells occurs primarily because of disturbed retrograde transport or axonal degeneration [22, 23]. Here, retrograde transport studies provided direct evidence that rHIgM22-mediated remyelination protected axon function in vivo. For efficient retrograde transport, axons have to not only be present but also functionally competent. Remyelination protected the function of spinal cord axons and led to approximately 35 % more FluoroGold-labeled brainstem neurons (Fig. 4b). Antibody-induced remyelination was sufficient to protect a significant number of axons from degeneration and promoted spinal cord axonal transport function. In addition, our data supports the concept that brainstem MRS may serve as a reliable surrogate marker in clinical trials designed to preserve or protect axons in the spinal cord.

The approach to remyelinate axons is considered to be protective as axon survival depends on trophic support from myelin [36]. Data in a mouse model of chronic demyelination indicates that mature glial cells produce various trophic factors that may aid in axonal survival long after myelin loss [37]. Positive correlations between inflammatory activity of MS lesions and axon damage provide support for immune-mediated axon degeneration [38] and suggest that axons may be naïve spectators in the surrounding inflammatory milieu during active demyelination. Even though some anti-inflammatory drugs may indirectly lead to a decrease in axonal damage during inflammatory CNS disease, no currently available drugs act directly on oligodendrocytes. The antibody described in this work, rHIgM22, does not bind to neurons, or supports neurite extension [13, 14], but acts on cells in the oligodendrocyte lineage to enhance endogenous remyelination. Prineas et al. recently showed that demyelination in human MS lesions followed the loss of caspase 3-positive apoptotic oligodendrocytes [39]. In this regard, we have evidence that rHIgM22 prevents apoptotic signaling and inhibits oligodendrocyte differentiation by Lyn [40]. This implies that rHIgM22-mediated remyelination is due to protection of oligodendrocyte progenitor cell (OPC) and oligodendrocytes rather than promotion of OPC differentiation [40]. rHIgM22 stimulates OPC proliferation in mixed glial cultures but not in purified OPCs. Stimulation of OPC proliferation by rHIgM22 depends on co-stimulatory astrocytic and/or microglial factors. We demonstrated that rHIgM22-mediated activation of PDGFalphaR is required for stimulation of OPC proliferation. Taken together, our data indicates that rHIgM22 lowers the PDGF threshold required for OPC proliferation and protection, which can ultimately result in remyelination of CNS lesions [41].

A number of lines of evidence support our conclusion that oligodendrocyte function has a major effect on axonal integrity. Oligodendrocytes play important roles in axon health and survival through glial–axonal signaling. However, axonal integrity requires more than glia signaling molecules. In Plp1-null mice lacking proteolipid protein (PLP), myelin assembly seems to be normal, whereas natural Plp1 mutants, such as jimpy and rumpshaker mice, exhibit dysmyelination caused by the toxicity of a misfolded protein [42]. Pelizaeus-Merzbacher disease (PMD) is an X-linked genetic disorder in which multiplication, deletion, or missense mutations of the *plp1* gene lead to elevated expression of proteolipid protein 1 (PLP1), and subsequently mild to severe dysmyelination-related symptoms [43]. In the later stages of disease, absence of compact myelin as well as widespread apoptosis of oligodendrocytes with superimposed axonal loss are evident. More than one gene may be responsible for the support of axons by myelinating glia. For example, 2′,3′-cyclic nucleotide 3′-phosphodiesterase (CNP), one of the earliest myelin-related proteins to be specifically expressed in differentiating oligodendrocytes, binds to RNA and tubulin and contributes to oligodendroglial process dynamics [44, 45]. Work done by Lappe-Siefke et al. indicated that CNP is essential for oligodendroglial functions in axonal support and myelination [46]. In addition, CNP is necessary for the formation of a normal inner tongue process of oligodendrocytes that myelinate small-diameter axons. Indeed, axonal degeneration in Cnp1 null mice is present very early in postnatal life [47]. Kang et al. demonstrated convincing oligodendrocyte dysfunction in both the motor cortex and spinal cord of amyotrophic lateral sclerosis (ALS) patients [48]. They hypothesized that direct impairment of oligodendrocyte function results in motor neuron vulnerability and faster disease progression. Beattie et al. demonstrated p75-dependent apoptotic death of oligodendrocytes in spinal cord injury [49]. Another group demonstrated that remyelination by neural precursor cells contributed to functional recovery in a rat model of spinal cord injury [50]. rHIgM22-mediated reduction of caspase 3 activity [40] on oligodendrocytes represents a novel therapeutic approach for many neurological diseases in which neurons and axons degenerate, such as ALS, PMD, other demyelinating and dysmyelinating diseases, and possibly spinal cord injury.

The above studies suggest that an improvement in oligodendrocyte function (remyelination) may protect axonal integrity. The goals of remyelination are neuroprotection, prevention of neuronal dysfunction, and maintenance of functional integrity of neurons and axons. The relevance of spontaneous remyelination in recovery of function was demonstrated in a cat model of demyelination with severe neurologic deficits [51] and in mice with focal demyelinated lesions resulting from ethidium bromide injection [52]. We demonstrated improved neurological function associated with spontaneous remyelination following extensive demyelination in the TMEV model [53]. This study strengthens our earlier results on remyelination but also demonstrates neuroprotection in the TMEV-induced model of inflammatory demyelinating disease where axon loss is progressive. Spontaneous remyelination in MS is generally limited [54, 55] with a block of adult OPC differentiation into myelin-producing oligodendrocytes as one potential reason [56]. Treatments to modulate this block to myelination are likely of clinical value. The use of an antagonist to LINGO-1 highlighted the importance of regulation of oligodendrocyte differentiation and myelination [57–59]. Correspondingly, treatment with anti-LINGO-1 antibody resulted in spinal cord remyelination and improved axonal integrity in MOG-induced experimental autoimmune encephalomyelitis [60] and lysolecithin-induced focal spinal cord demyelination in rats [61]. In another study, retinoid acid receptor agonists (rexinoids) were used to successfully stimulate OPC differentiation and promote remyelination of demyelinated cerebellar slices and toxin-induced demyelinated lesions in vivo [62].

Toxicity of the drug in vivo is a major issue for many treatments to enter the market. The safety and tolerability of single ascending dose of rHIgM22 was recently investigated in a phase 1, multicenter, double-blind, randomized, placebo-controlled, dose-escalation study in patients with MS [17]. No dose-limiting toxicities or serious treatment-emergent adverse events were reported at any rHIgM22 dose level [63]. rHIgM22 was measurable in the cerebrospinal fluid (CSF) in all patients at day 2 and in a proportion of patients at day 29 following administration of a single dose. This observation strongly supports the hypothesis that blood–brain/blood–CSF barrier is permeable to CNS-modulating drugs across, including large molecules such as IgM. Indeed, molecules as large as IgM are present in normal CSF at approximately 1/1000 of their serum concentration [64]. In the past, we used non-invasive T2-weighted MRI studies and showed that USPIO-labeled rHIgM22 antibody associated with a decreased spinal cord lesion load [65]. No IgM accumulation in the CNS was apparent in non-infected animals or animals without demyelination. Therapeutic approaches that aim to boost intrinsic properties of progenitor cells or to supply progenitors by cell transplantation approaches seek at promoting remyelination and also to inhibit neurodegeneration. However, a key question is whether the antibody- or cell-based remyelination strategies offer a permanent solution. Further studies to understand the mechanisms and complex molecular factors that regulate remyelination should help answer these concerns.

CNS, central nervous system; MRS, magnetic resonance spectroscopy; MS, multiple sclerosis; NAA, *N*-acetyl-aspartate; PPMS, primary progressive multiple sclerosis; rHIgM22, recombinant human immunoglobulin M 22; RRMS, relapsing remitting multiple sclerosis; SPMS, secondary progressive multiple sclerosis; SJL, Swiss Jim Lambert; TMEV, Theiler’s murine encephalomyelitis virus
